# Association Between Colleague Violence and the Professional Image of Nursing and Career Decisions Among Nursing Students: A Cross‐Sectional Study

**DOI:** 10.1111/jan.16791

**Published:** 2025-02-03

**Authors:** Sebahat Kuşlu, Emine Karacan, Soner Berşe

**Affiliations:** ^1^ Department of Nursing, Faculty of Health Sciences Gaziantep Islam Science and Technology University Gaziantep Turkey; ^2^ Department of Health Care Services, Vocational School of Health Services Gaziantep Islam Science and Technology University Gaziantep Turkey; ^3^ Faculty of Health Sciences Gaziantep University Gaziantep Turkey

**Keywords:** career decision, colleague violence, cross‐sectional study, nursing image, nursing students

## Abstract

**Aim:**

To examine how colleague violence affects professional image and career decisions among nursing students.

**Design and Setting:**

This cross‐sectional study was conducted from February 1 to March 1, 2023, at two public universities in Turkey.

**Sample and Methods:**

All nursing students who met the inclusion criteria and voluntarily agreed to participate were included without any special sample calculation, and a final sample of 357 students was obtained. Data were collected between February 1 and March 1, 2023. Data were collected using a survey form that included questions on sociodemographic characteristics, as well as three scales: the Exposure to Colleague Violence Scale (ECVS) scale, the Image of Nursing Profession Scale (INPS) scale, and the Career Decision Scale (CDS). Data analysis included descriptive statistics, *t*‐tests, ANOVA, Pearson correlation, and regression analyses.

**Results:**

The mean ECVS score was 45.43 (20.80), the mean INPS score was 147.15 (13.51), and the mean CDS score was 79.67 (17.34). A weak negative correlation was found between colleague violence and nursing image, a weak positive correlation between colleague violence and career decision, and a moderate negative correlation between nursing image and career decision.

**Conclusion:**

This study highlights the negative impact of exposure to colleague violence on nursing students, affecting both their professional image and career decision‐making. Greater exposure to colleague violence correlates with more negative perceptions of the nursing profession and increased uncertainty in career choices. Implementing targeted interventions to reduce and prevent colleague violence, especially in clinical practice settings, is essential for promoting a positive professional image and supporting informed career decisions among nursing students.

**Implications for the Profession and Patient Care:**

Addressing and reducing colleague violence among nursing students can enhance their professional image and decision‐making regarding their careers, which, in turn, may lead to improved patient care and more significant long‐term commitment to the nursing profession.

**Reporting Method:**

This study adhered to the STROBE (Strengthening the Reporting of Observational Studies in Epidemiology) guidelines for cross‐sectional studies.

**Patient or Public Contribution:**

There was no patient or public involvement in this study.

**Summary**

- What problem did the study address?
  ○ This study examined the issue of colleague violence among nursing students, focusing on its effects on their professional image and career‐related decisions.
- What were the main findings?
  ○ Increased exposure to colleague violence diminishes the perceived professional image of nursing and reduces career decisiveness.
  ○ Colleague violence adversely affects nursing students' professional self‐image and their career‐related decisions.
- Where and on whom will the research have an impact?
  ○ The findings have implications for nursing education and clinical practice, particularly for nursing students and educators in Turkey. This study underscores the importance of implementing targeted interventions to reduce colleague violence in clinical settings. Educators can help mitigate the risk of colleague violence by integrating programmes into the nursing curriculum that focus on conflict resolution, emotional resilience, effective communication, and coping strategies. These interventions will not only support nursing students' professional growth but also foster a more supportive and collaborative clinical environment.
- What does this paper contribute to the wider global clinical community?
  ○ Highlights the negative impact of colleague violence on the professional development of nursing students
  ○ Emphasises the urgent need for interventions to prevent colleague violence in clinical settings.
  ○ Offers insights that can enhance nursing education and student support programmes.
  ○ Provides specific recommendations for improving nursing education by incorporating targeted interventions to prevent colleague violence, including workshops on conflict resolution and communication skills and strengthened mentorship programmes to support nursing students in clinical practice.

## 1 Introduction

Colleague violence, which can occur both in educational settings and professional life, involves aggression perpetrated by one colleague towards another. It can manifest in various forms, including verbal and emotional abuse as well as physical attacks and represents a significant source of occupational stress (Koc and Batkin 2016). Colleague violence in nursing often manifests through behaviours such as constant criticism, derogatory comments, humiliation, undue pressure, loud or aggressive speaking, groundless accusations, ignoring or isolating others, sarcasm, scapegoating and excessive workload demands (Gungor and Tosunoz 2024) Colleague violence in the workplace can lead to absenteeism, high turnover, increased stress, anxiety, emotional distress and, in severe cases, post‐traumatic stress disorder (Fernandez‐Gutierrez and Mosteiro‐Diaz 2021).

Exposure to colleague violence among nursing students negatively affects their motivation to learn, their perceptions of the profession, professional growth, career commitment, the quality of their clinical training, and their interpersonal relationships with team members, patients, and patients' families (Gungor and Tosunoz 2024). Consequently, colleague violence is regarded as one of the most significant sources of stress within professional settings. In a study conducted in Hong Kong, 37% of nursing students reported experiencing violence during clinical placements (Cheung et al. 2019). Another study found that 69.2% of nursing students had encountered some form of violence, primarily perpetrated by teachers or classmates (Maffissoni et al. 2021). A study from Iran found a workplace violence rate of 73% among nursing students (Samadzadeh and Aghamohammadi 2018). In a systematic review and meta‐analysis by Mohammed et al., 55.1% of nursing students reported exposure to workplace violence, with 24.2% of incidents perpetrated by nurses (Mohamed et al. 2024). Similarly, a study from Turkey reported that 40.2% of nursing students experienced violence during clinical practice, with 66.7% of perpetrators being nurses (Tonkus and Coskun 2021).

Career progression involves advancing within a profession, gaining status and earning respect. Both personal and environmental factors influence career decisions. Personal factors include gender, age, and socioeconomic status, while environmental factors encompass the workplace setting, working hours, communication with patients and their families and interactions with colleagues (Tengiz and Babaoglu 2020). In this context, colleague violence stands out as a significant environmental factor influencing students' career decisions.

The nursing image reflects how nurses perceive themselves and their colleagues, as well as how they are viewed by society (Kuslu et al. 2022). Perceptions of colleagues are shaped by factors such as communication, harmony, shared experiences, and solidarity among colleagues. Positive communication, harmony, and solidarity among colleagues are reported to enhance an individual's professional image, foster teamwork and autonomy and reduce instances of aggression and violence (Göktepe et al. 2020).

Nursing students are particularly vulnerable to colleague violence in healthcare settings due to their inexperience. During clinical placements, nursing students provide care under the supervision and guidance of experienced nurses. It is crucial for nurses to adopt a supportive attitude towards nursing students during these placements, as this fosters a sense of safety within the healthcare team and enhances professional satisfaction by ensuring a positive clinical experience (Gungor and Tosunoz 2024). A review of the literature reveals numerous studies addressing colleague violence in nursing (Karatepe Kuscu, Yuce Ozcan, and Atik 2020), the professional image of nursing (Totur Dikmen et al. 2022), and career decision‐making (Tengiz and Babaoglu 2020). However, no existing research has explored the intersection of these three concepts simultaneously. This study aims to fill that gap by examining how exposure to colleague violence affects nursing students' professional image and career decisions. The insights gained are expected to contribute significantly to the literature and to inform strategies for preventing violence and mitigating its impact in both clinical and academic settings.

## 2 The Study

### 2.1 Aim

The aim of the study was to determine the impact of colleague violence on nursing image and career decision among nursing students.

### 2.2 Research Hypotheses

Based on the aims of this study, the following research hypotheses were proposed:

Hypothesis 1 *Nursing students' exposure to colleague violence significantly affects their perception of the nursing image*.

Hypothesis 2 *Nursing students' exposure to colleague violence significantly affects their career decisions*.

## 3 Methods

### 3.1 Design

A cross‐sectional research design was used to examine how exposure to colleague violence affects nursing students' perceived professional image and career decisions.

### 3.2 Study Setting and Sampling

The study was conducted from February 1 to March 1, 2023, at two public universities located in Gaziantep province, Turkey. The study population consisted of undergraduate students from nursing departments of these universities. Without employing sampling calculations, all nursing students who met the inclusion criteria and voluntarily agreed to participate were included, resulting in a total sample of 357 students. To assess the adequacy of this sample size, a post hoc power analysis was conducted using G*Power 3.1.9.7 software (Faul et al. 2020). The analysis was based on a two‐tailed test with a significance level of *α* = 0.05 and the observed correlation coefficient (*r* = −0.307) in this study between ECVS and INPS. The statistical power was calculated as 99.99% (Power = 0.99), confirming that the sample size was sufficient to detect significant effects. Participants were recruited from both universities and represented all academic years (1st, 2nd, 3rd and 4th years) to ensure a diverse sample and minimise potential bias.

Nursing students were included if they were at least 18 years old, enrolled in the Nursing Departments of state universities in Gaziantep province, voluntarily agreed to participate, and had no health or communication problems that could impede their ability to answer the survey questions.

### 3.3 Data Collection Tools

The data were collected through face‐to‐face interviews using a survey form created by the authors on Google Forms. The survey included questions on socio‐demographic characteristics and three scales developed and validated in Turkish: the Exposure to Colleague Violence Scale (ECVS), the Image of Nursing Profession Scale INPS and the Career Decision Scale (CDS). A link to the survey was sent to each participant for data collection. To ensure participant anonymity, the survey form did not include identifying information such as student's names or ID numbers, thus minimising information bias and encouraging honest responses. Before data collection, the survey procedure was thoroughly explained to the participants, with an emphasis on the importance of completing all sections. Additionally, an online survey tool (Google Forms) was used, which automatically prompted participants to complete any unanswered questions before submitting the survey. As a result, all participants completed the full survey in full, and no missing data was recorded.

#### 3.3.1 Socio‐Demographic Characteristics

The survey collected data on various socio‐demographic characteristics of the participants, including age, sex, academic year, household income, primary place of residence and reasons for choosing the nursing department.

#### 3.3.2 Exposure to Colleague Violence Scale (ECVS)

The ECVS, developed by Yilmaz Bahadir, Ata, and Uyumaz (2020), measures the extent of colleague violence experienced by nursing students from their colleagues. The scale consists of 22 items divided into two subscales: ‘the exposure to verbal/psychological violence’ and ‘the impact of violence on physical and mental health’. Each item is scored on a 5‐point Likert scale from 1 (Strongly Disagree) to 5 (Strongly Agree), with no reverse‐scored items. Total scores range from 22 to 110, with higher scores indicating a greater level of exposure to colleague violence in clinical settings. The intercept point of the scale is 66 points. The scale showed a Cronbach's alpha of 0.94 (Yilmaz Bahadir, Ata, and Uyumaz 2020), while in the current study, Cronbach's alpha was 0.97.

*Example item for the* ‘*exposure to verbal*/*psychological violence subscale*’: ‘I am subjected to hurtful comments’.

*Example item for the impact of violence on physical and mental health subscale*: ‘I don't want to go to the internship because of your behavior towards me’.

#### 3.3.3 Image of Nursing Profession Scale (INPS)

Developed by Dost and Bahcecik (2015), the INPS assesses the perception of the nursing profession across six domains: Professional Qualifications, Working Conditions, Gender, Education, Professional Status and External Appearance. This 42‐item scale is rated on a 5‐point Likert scale from 1 (Strongly Disagree) to 5 (Strongly Agree), with total scores ranging from 42 to 210. A score between 42 and 75 indicates a very poor perception of the nursing profession's image; scores between 76 and 109 reflect a weak perception; 110–143 suggests a moderate perception; 144–177 represents a good perception; and scores between 178 and 210 indicate a very strong perception A Cronbach's alpha of 0.88 was reported for the INPS (Dost and Bahcecik 2015), and in this study, the Cronbach's alpha was 0.75.

Example items for each of the INPS domains are provided below:

*Professional qualifications*: ‘Nurses are the healthcare workers who spend the most time with patients’.

*Working conditions*: ‘Nursing is a profession with a high risk of exposure to violence’.

*Gender*: ‘Negative perceptions of nursing are decreasing as more men join the profession’.

*Education*: ‘Nursing requires constant learning’.

*Professional status*: ‘Nursing is a profession respected by other healthcare professionals’.

*Physical appearance*: ‘Nurses are well groomed’.

#### 3.3.4 Career Decision Scale (CDS)

The CDS, developed by Yusupu (2015), measures career decision‐making across three subscales: Conscious Career Decision, Unconscious Career Decision, and Environmental factors. The tool consists 30 items, each rated on a 5‐point Likert scale from 1 (Not Suitable for Me at All) to 5 (Completely Suitable for Me). All items in the Conscious Career Decision subscale are reverse‐coded. Total scores range from 30 to 150, with higher scores indicating indecisiveness and lower scores indicating decisiveness. The intercept point of the scale is 90 points. The CDS showed a Cronbach's alpha of 0.91 (Yusupu 2015). In this study, the Cronbach's alpha was 0.90.

Example items for each of the CDS subscales are presented below:

*Conscious career decision*: ‘I'm making plans to achieve my career goals’.

*Unconscious career decision*: ‘When making my career decision, I was so confused that I just went with the flow and ended up choosing my current career.’

*Environmental factors*: ‘I chose my career because my family wanted me to, so the courses don't really interest me.’

### 3.4 Data Analysis

The data were analysed using IBM SPSS version 23.0 (IBM Corp 2015, Armonk, NY, USA). The analyses revealed that the skewness‐kurtosis values of the scales ranged between −1.5 and + 1.5, indicating a normal distribution in line with the literature (Tabachnick and Fidell 2013; Kline 2015). The values were 0.887/0.314 for ECVS, −0.548/1.091 for INPS and −0.794/0.246 for CDS. Descriptive statistics, including frequencies, percentages, means and standard deviations, were used to summarise the socio‐demographic characteristics of the participants. Since the data followed a normal distribution, parametric statistical methods were applied. Independent samples *t*‐tests were conducted to compare two groups, while ANOVA was used for comparisons among three or more groups. Pearson correlation analysis was performed to examine relationships between numerical variables, and regression analysis was conducted to assess the impact of colleague violence on the nursing image and career decisions. A significance level of *p* < 0.05 was used for all analyses. There is no missing data in this study; all analyses were performed using the full data set.

### 3.5 Ethical Considerations

Ethical approval for the study was obtained from the Institutional Review Board of Gaziantep Islam Science and Technology University (December 9, 2022; No: 2023/187). The research was conducted in accordance with ethical standards. Additionally, informed consent was obtained from all participants after they were fully informed about the study's purpose, methodology, potential benefits and risks. Participants were also informed that they could withdraw from the study at any time without consequence.

## 4 Results

A total of 800 students were invited to the survey and 357 students participated. This represents a response rate of 44.62%.

### 4.1 Characteristics of the Sample

The mean age of the nursing students participating in the study was 20.56 (1.59) years. The majority of participants were female (74.8%), with first‐year students comprising 46.5% of the sample. 37.3% reported a household income ranging from 8001 to 12,000 TL and 56.3% had mostly lived in the city center. Additionally, 56.6% of the student reported choosing nursing profession due to its perceived ease of employment (Table 1).

**TABLE 1:** The association between socio‐demographic characteristics of nursing students and their total scores on ECVS, INPS and CDS.

Socio‐demographic characteristics (*n* = 357)	*r*/*p*		ECVS		INPS		CDS	
Age (mean/SD): 20.56/1.59			0.04/0.36		−0.01/0.78		−0.05/0.30	
	*n*	%	Mean	SD	Mean	SD	Mean	SD
Sex								
Female	267	74.8	46.52	20.68	147.77	13.68	78.77	17.54
Male	90	25.2	42.20	20.95	145.33	12.87	82.34	16.53
Statistical analysis (*t*/*p*)			1.71/0.08		1.48/0.13		−1.69/0.09	
Academic year								
1	166	46.5	43.16	21.77	147.43	13.61	80.34	16.46
2	76	21.3	47.57	19.78	145.53	15.87	81.02	19.34
3	108	30.3	46.41	19.47	148.01	11.83	77.37	17.47
4	7	2.0	60.85	22.42	144.71	5.67	84.57	10.73
Statistical analysis (*F*/*p*)			2.31/0.07		0.60/0.61		1.05/0.36	
Household Income								
0–4000 TL	38	10.6	46.97	20.80	146.97	16.11	81.76	18.92
4001–8000 TL	108	30.3	45.07	20.89	146.54	13.08	81.66	17.20
8001–12,000 TL	133	37.3	44.02	20.19	147.83	14.30	76.93	17.44
More than 12,000 TL	78	21.8	47.60	21.87	146.93	11.35	80.56	16.26
Statistical analysis (*F*/*p*)			0.56/0.63		0.19/0.90		1.84/0.13	
Predominant place of residence								
City center	201	56.3	47.90	22.58	146.99	14.12	78.44	17.55
District center	90	25.2	43.24	18.33	146.96	14.07	79.55	17.98
Village/town	66	18.5	40.90	17.18	147.92	10.68	83.57	15.39
Statistical analysis (*F*/*p*)			3.52/0.03		0.13/0.87		2.18/0.11	
Reasons for choosing the nursing department								
At the insistence of family	50	14.0	55.88	24.15	140.80	13.68	90.20	10.87
Due to perceived ease of employment and undergraduate education	202	56.6	45.16	19.89	146.56	13.05	81.67	15.32
Because of the positive social image	95	26.6	40.55	19.26	151.92	13.38	69.49	19.45
Because I love it	10	2.8	45.00	19.33	145.50	7.18	83.40	15.50
Statistical analysis (*F*/*p*)			6.21/**< 0.001**		8.29/**< 0.001**		21.17/**< 0.001**	
Total $\bar{x}$ ± SD			45.43	20.80	147.15	13.51	79.67	17.34
Min–Max			22–110		87–178		30–123	

Abbreviations: CDS, Career Decision Scale; ECVS, Exposure to Colleague Violence Scale; *F*, coefficient of one‐way ANOVA; INPS, Image of Nursing Profession Scale; *p*, significance level; *r*, coefficient of correlation; SD, standard deviation; *t*, coefficient of independent sample *t*‐tests.

*Note:* Bold values indicates “*weak correlation”, “**moderate correlation”.

### 4.2 Association Between Socio‐Demographic Characteristics and Scale Scores

Several patterns emerged when examining the association between the students' socio‐demographic characteristics and their scale scores. Firstly, the students who mostly resided in urban areas exhibited higher ECVS scores compared to those living in rural areas. Secondly, the students who chose the nursing department at the insistence of their families showed higher ECVS scores than those who chose it based on perceived ease of employment and the nature of undergraduate education. This group also had lower INPS scores but higher mean CDS scores. Furthermore, those who chose nursing at the urging of their families showed higher ECVS and CDS scores compared to those who were drawn to the profession due to its positive social image, though their INPS scores were lower. Lastly, the students who selected nursing based on perceived ease of employment and the nature of undergraduate education exhibited higher INPS and CDS scores. These differences were statistically significant (*p* < 0.05), as shown in Table 1.

The mean ECVS score for the nursing students was 45.43 (20.80), the mean INPS score was 147.15 (13.51) and the mean CDS score was 79.67 (17.34) (Table 1).

### 4.3 Correlation Between Scale Scores

A weak negative correlation was found between ECVS and INPS scores, while a weak positive correlation was observed between ECVS and CDS scores. Additionally, a moderate negative correlation was found between INPS and CDS scores (*p* < 0.05) (Table 2).

**TABLE 2:** Correlation between ECVS, INPS and CDS (*n* = 357).

Scales	ECVS	INPS	CDS
ECVS			
*r*		−0.307	0.392
*p*		< 0.001*	< 0.001*
INPS			
*r*			−0.472
*p*			< 0.001**

Abbreviations: CDS, Career Decision Scale; ECVS, Exposure to Colleague Violence Scale; INPS, Image of Nursing Profession Scale; *p*, significance level, *weak correlation, **moderate correlation; *r*, coefficient of correlation.

*Note:* Bold values indicates “*weak correlation”, “**moderate correlation”.

### 4.4 Findings on the Effect of Colleague Violence on Nursing Image and Career Decision

Regression analysis was conducted to examine the impact of exposure to colleague violence on‐nursing image and career decision. The results showed that exposure to colleague violence significantly affects both nursing image (*p* < 0.001) and career decision (*p* < 0.001). Specifically, colleague violence explained 9.5% of the variance in nursing image and 15.4% of the variance in career decision (Table 3).

**TABLE 3:** Univariate regression analysis on the predictive power of ECVS on INPS and CDS (*n* = 357).

Results of regression analysis						Results regarding the validity of the established model			
Independent variable	Dependent variable	*β*	*t*	*p*	Confidence Interval (LL/UL)	*F*	*p*	*R* ^2^	Durbin‐Watson
ECVS	Constant	156.227	95.348	**< 0.001**	153.004/159.449	37.049	< 0.001	0.095	1.810
	INPS	−0.200	−6.087	< 0.001*	−0.264/−0.135				
ECVS	Constant	176.458	59.377	**< 0.001**	170.613/182.302	64.429	< 0.001	0.154	2.031
	CDS	0.327	8.027	< 0.001*	0.247/0.407				

Abbreviations: *β*, standardized regression coefficient; CDS, Career Decision Scale; Durbin‐Watson, coefficient of autocorrelation; ECVS, Exposure to Colleague Violence Scale; *F*, overall significance level of the model; INPS, Image of Nursing Profession Scale; LL, lower limit; p, significance level; *R* ^2^, coefficient of determination; UL, upper limit.

*Note:* Bold values indicates “*weak correlation”, “**moderate correlation”.

## 5 Discussion

Although colleague violence occurs across various professions, it is particularly prevalent in nursing due to the high levels of stress associated with frequent patient interactions, shift work, long working hours, and insufficient materials and equipment (Bernardes et al. 2020). Nursing students, especially during the early stages of their careers, nursing students are often excluded or subjected to violence by their colleagues, particularly in clinical settings (Gungor and Tosunoz 2024). Such experiences can negatively affect their career choices and their perception of the nursing profession.

This study tested two hypotheses, both of which were supported by the results:

Hypothesis 1 *‘Nursing students' exposure to colleague violence significantly affects their perception of the nursing image’. This hypothesis was accepted since the results revealed a significant negative correlation between exposure to colleague violence and nursing students' perceptions of the nursing profession (r = −0.307, p < 0.05). As exposure to colleague violence increased, students' perceptions of the nursing profession became more negative.*

Hypothesis 2 *‘Nursing students' exposure to colleague violence significantly affects their career decisions’. This hypothesis was also accepted. A significant positive correlation was observed between exposure to colleague violence and career indecision (r = 0.392, p < 0.05), indicating that greater exposure to violence is associated with greater uncertainty regarding career choices.*

These findings highlight the detrimental effects of colleague violence on both the professional image and career decision‐making processes of nursing students. They emphasise the critical need for interventions to reduce such violence in clinical and educational settings, as it poses risks to both professional identity formation and career commitment.

In the current study, nursing students reported relatively low levels of exposure to colleague violence, maintained a positive image of the nursing profession, and demonstrated clarity and determination in their career choices. These findings are consistent with those of similar studies (Cao et al. 2023; Olgun and Kaptan 2022). To mitigate colleague violence, nursing education programmes should incorporate targeted training on recognising and addressing such behaviours, developing effective coping strategies, and fostering interventions to prevent bullying. Emotional resilience training has also proven effective in helping nursing students and professionals cope with stress and prevent burnout, which is often associated with violent encounters (Sukut and Ayhan 2022). By integrating these evidence‐based approaches into the nursing curriculum, students will be better equipped to navigate interpersonal challenges, contribute to a more supportive environment and ultimately enhance patient care.

The results of this study showed that nursing students' exposure to peer violence negatively affected both their image of the profession and their career decisions. In particular, as students' exposure to colleague violence increased, their perceptions of nursing became more negative and they became more uncertain about their career paths. This may affect students' academic performance and self‐perception. Because research has shown that exposure to workplace violence is associated with lower levels of academic engagement and self‐confidence among nursing students (Hung et al. 2019). Moreover, as workplace violence is a contributing factor to high turnover rates in healthcare settings (Liu et al. 2018), such exposure may lead students to consider leaving the profession. In addition to the impact on career decisions, peer violence can lead to stress, distress and depression, all of which negatively impact patient care. Studies have shown that psychological distress caused by workplace violence can impair nurses' ability to provide quality care and ultimately affect patient outcomes (Wilson 2016). Similarly, in a study conducted by Kirbas and Kahriman (2023), it was found that difficulties encountered during clinical placements led students to consider leaving the nursing profession (Kirbas and Kahriman 2023). These findings emphasise the importance of addressing workplace violence in nursing education and practice to prevent negativities.

Violence has been shown to have a negative impact on professional development, motivation and self‐efficacy (Koc and Batkin 2016; Wilson 2016). The occurrence of colleague violence during a critical period when students are shaping their professional identities in clinical settings suggests that it may have lasting consequences. Research indicates that experiencing violence undermines students' trust in their professional role models and decreases their motivation to remain in the profession (Hung et al. 2019). Studies have also found that students who struggle to cope with violence are at an increased risk of high stress, anxiety, and burnout, which can negatively impact both their well‐being and the quality of patient care (Mento et al. 2020). Given these findings, strategic interventions in both educational and clinical settings are crucial to prevent colleague violence. Incorporating conflict management, effective communication, and emotional resilience training into nursing curricula may help mitigate the negative impact of such experiences. Research has demonstrated that training nursing students in conflict resolution techniques leads to improved coping skills and lower levels of stress and aggression in clinical settings (Choi and Ahn 2021). Furthermore, implementing zero‐tolerance policies in clinical settings and encouraging students to report incidents of violence are essential steps in creating a safer learning environment. Mentorship programmes can also play a key role in fostering a positive image of the nursing profession and supporting students in remaining committed to their careers. These programmes have been shown to improve students' professional identity and reduce feelings of isolation, enhancing both their perceptions and the overall image of the nursing profession (Rudin and Ludin 2018).

In a study examining the nature, extent and contributing factors of workplace violence experienced by Australian nursing students during clinical placements, it was reported that tolerance for and normalisation of violence were among the factors that contributed to the persistence of workplace violence (Johnston et al. 2024). To improve the public image of nursing and encourage future nurses to embrace the profession, it is crucial that such violent behaviours be neither accepted nor tolerated, whether among nurses or in any other professions. Moreover, establishing mentorship programmes and supportive clinical environments can help reduce the incidence of colleague violence. Mentors can provide guidance, support, and model professional behaviour for students, which may improve their professional image and career decisiveness (Rudin and Ludin 2018). Nursing faculties and clinical institutions should collaborate to develop policies that discourage colleague violence and promote a culture of respect, collaboration and professionalism.

The study found that students residing in urban areas were more exposed to colleague violence compared to those living in rural areas. It is believed that individuals living in urban areas tend to be more active and assertive than those in rural areas. It was suggested that individuals with these personality traits are more likely to be exposed to colleague violence (Barroso‐Corroto et al. 2024). Thus, it is important to identify the personality traits of those engaging in mobbing behaviour and address any related issues (Həsənli 2024). Furthermore, students coming from geographically disadvantaged areas may benefit from empowerment initiatives aimed at enhancing their knowledge, skills and professional practices.

Career choice is a significant milestone in a person's life, shaped by many factors. Choosing a profession willingly contributes to better professional integration, greater job satisfaction and more positive relationships with colleagues (Ozdelikara, Agacdiken, and Aydin 2016). In a study aiming to explore nursing students' views on career choice, Ozdelikara, Agacdiken, and Aydin (2016) found that students who chose the nursing profession willingly were more vocationally suited than those who did not. In the current study, nursing students who chose the profession under family pressure were more susceptible to colleague violence than those who chose it based on perceived career prospects, the ease of undergraduate education or the positive social perception of the profession. Drawing from the literature and the findings of this study, to increase the rate of voluntary career choice in nursing, both practitioners and community should work to professionalise the field, enhance its visibility and create a positive image. It is recommended that career decisions be based on individual's talents, preferences and independent judgement.

Nursing students who choose the profession based on perceived ease of employment and the educational path tend to have a more positive professional image than those who choose it solely due to family insistence. As such, when students consider their own educational career aspirations in choosing nursing, it suggests that they are aligning their career decisions with personal desires, which fosters more optimistic and positive views of the profession. As a result, satisfaction with the profession and the nursing image are likely to improve. As professional satisfaction grows, individuals are more likely to embrace the profession, assume a professional identity and navigate their careers more confidently.

Another finding from this study revealed that students who chose nursing at the insistence of their families experienced greater career indecision compared to those who chose it for other reasons. This suggests that students' who make career choices without informed and independent decision‐making may become more dependent and uncertain about their professional paths. This indecision negatively affects their adaptation to the profession, their professional image and their relationships with colleagues (Stead, LaVeck, and Rúa 2021).

Those who choose the nursing profession because of its positive image in society tend to perceive the profession more positively compared to those who choose it due to family pressure. Career choice is a critical stage where individuals express their thoughts and assert their independence. Choosing a profession willingly leads to greater job satisfaction, enhanced professional identity and a positive professional image (Cakir and Buldukoglu 2020). Therefore, it can be inferred that individuals who are drawn to nursing because of its positive social image likely have higher professional self‐esteem and independence than those who pursue it under family influence. As a result, they are likely to view the nursing profession more favourably.

### 5.1 Limitations

Several limitations should be considered when interpreting the findings of this study. First, the data collection was limited to nursing students from only two universities in Gaziantep, Turkey. This geographical limitation prompts inquiries regarding the applicability of the findings to nursing students from diverse regions or attending various educational institutions. Additionally, the analysis of socio‐demographic factors could have been expanded to include other potential interactive variables, such as students' ethnicity, socioeconomic status and cultural backgrounds. A broader examination of such variables could offer deeper insights into colleague violence and help develop more effective strategies for addressing this issue.

Another limitation of the study is the low response rate of 44.62%. This may limit the generalizability of the results to all nursing students and may create uncertainty about whether the findings obtained from the sample are applicable to a larger population of nursing students. Given these limitations, caution should be taken when generalising the study's conclusions and future research should be designed to address these limitations.

## 6 Conclusions

The findings revealed that while nursing students reported low levels of exposure to colleague violence, their perceptions of the nursing profession were generally positive, and they were determined in their career choices. In addition, it was observed that exposure to colleague violence negatively affected students' perceptions of the profession and career decisions. Based on these findings, educational programmes should be implemented to help nursing faculty students and healthcare professionals to recognise colleague violence, develop coping strategies and address bullying behaviours. In this context, conflict management and effective communication skills courses can be integrated into the nursing curriculum. Nursing students should be offered programmes that focus on conflict management, effective communication skills, and emotional resilience. Mentoring programmes should be established to provide students with professional support in managing colleague violence during clinical placements. By guiding students, mentors can model professional behaviour and increase students' professional commitment. Zero‐tolerance policies against violence should be introduced in nursing faculties and healthcare institutions, with clear guidelines to support students in navigating challenging clinical situations. These guidelines should include clear guidelines that support the reporting of incidents of violence and facilitate students to cope with the challenges they face in the clinical setting. No nurse should accept or tolerate bullying, lateral violence, or incivility in the workplace. Nurses should establish policies against bullying, report abusive behaviour and be encouraged to report incidents of incivility in the workplace. Additionally, measures should be taken to protect employees who experience violence in the workplace and to ensure appropriate investigation processes. Standard operating procedures should be developed for the effective and impartial investigation of reported incidents of bullying and violence. Psychological support and counselling services should be provided for both victims and witnesses after the incident. Interventions aimed at enhancing student resilience should be incorporated into nursing curricula. Both nurses and nursing students should be encouraged to engage more actively in professional organisations, particularly during the early stages of their careers. These organisations can develop joint policies and advocate for solutions to problems such as workplace violence and bullying. Student nurses, as the future of the profession, must receive training that enhances their skills and practice. Professional organisations, seminars, conferences and workshops should be organised to provide nurses and students with the opportunity to learn current knowledge and best practice. Future research should explore nursing students' perspectives on potential solutions to colleague violence. For this purpose, one‐to‐one interviews and focus group studies should be conducted with students to investigate their experiences and suggestions for solutions to colleague violence. Comprehensive questionnaires and scales can be prepared to evaluate student opinions on strategies to prevent colleague violence. Simulations involving cases of violence that may be encountered in clinical practice can be organised. These trainings can be effective in enabling students to learn how to react in challenging situations.

## Author Contributions

All authors have agreed on the final version and meet at least one of the following criteria: (1) Substantial contributions to conception and design, acquisition of data, or analysis and interpretation of data, (2) Drafting the article or revising it critically for important intellectual content.

## Ethics Statement

Ethics Committee for Non‐Interventional Clinical Research of a university (Decision no: 187.22.12).

## Consent

Informed consent was obtained from all individuals participating in the research after they were provided with detailed explanations of the purpose, methodology, potential benefits and risks of the study.

## Conflicts of Interest

The authors declare no conflicts of interest.

## Supporting information


**Data S1.** Supporting Information.

## Data Availability

Research data are not shared.

## References

[jan16791-bib-0001] Barroso‐Corroto, E. , J. A. Laredo‐Aguilera , A. I. Cobo‐Cuenca , and J. M. Carmona‐Torres . 2024. “Experiences of Nursing Students Who Are Victims of Dating Violence: A Qualitative Study.” BioMed Central Nursing 23, no. 1: 28. 10.1186/s12912-023-01688-w. 38195560 PMC10775457

[jan16791-bib-0002] Bernardes, M. L. G. , M. E. Karino , J. T. Martins , C. V. C. Okubo , M. J. Q. Galdino , and A. A. O. Moreira . 2020. “Workplace Violence Among Nursing Professionals.” Revista Brasileira de Medicina Do Trabalho 18, no. 3: 250. 10.47626/1679-4435-2020-531. PMC7879475 33597974

[jan16791-bib-0003] Cakir, C. , and K. Buldukoglu . 2020. “The Effect of Cognitive Distortions on Nurses' Professional SelfEsteem and Nursing Perception.” Ankara Journal of Health Sciences 9, no. 1: 193–206. 10.46971/ausbid.669614.

[jan16791-bib-0004] Cao, J. , H. Sun , Y. Zhou , A. Yang , X. Zhuang , and J. Liu . 2023. “Nursing students' Experiences of Workplace Violence Based on the Perspective of Gender Differences: A Phenomenological Study.” BioMed Central Nursing 22, no. 1: 387. 10.1186/s12912-023-01551-y. 37853431 PMC10583471

[jan16791-bib-0005] Cheung, K. , S. S. Ching , S. H. N. Cheng , and S. S. M. Ho . 2019. “Prevalence and Impact of Clinical Violence Towards Nursing Students in Hong Kong: A Cross‐Sectional Study.” BMJ Open 9, no. 5: e027385. 10.1136/bmjopen-2018-027385. PMC6530330 31101698

[jan16791-bib-0006] Choi, H. , and S. Ahn . 2021. “Effects of a Conflict Resolution Training Program on Nursing Students: A Quasi‐Experimental Study Based on the Situated Learning Theory.” Nurse Education Today 103: 104951. 10.1016/j.nedt.2021.104951. 34015679

[jan16791-bib-0007] Dost, A. , and A. N. Bahcecik . 2015. “Developing A Scale for the Image of Nursing Profession.” Journal of Asian Earth Sciences 1, no. 2: 51–59. 10.5222/jaren.2015.051.

[jan16791-bib-0008] Faul, F. , E. Erdfelder , A. Buchner , and A.‐G. Lang . 2020. “Statistical Power Analyses Using G*Power 3.1: Tests for Correlation and Regression Analyses.” Behavior Research Methods 41, no. 4: 1149–1160. 10.3758/BF03193146. 19897823

[jan16791-bib-0034] Fernández‐Gutiérrez, L. , and M. P. Mosteiro‐Díaz . 2021. “Bullying in Nursing Students: A Integrative Literature Review.” International journal of mental health nursing 30, no. 4: 821–833. 10.1111/inm.12854. 33848043

[jan16791-bib-0036] Göktepe, N. , B. Yalçın , E. Türkmen , Ü. Dirican , and M. Aydın . 2020. “The Relationship Between Nurses’ Work‐Related Variables, Colleague Solidarity and Job Motivation.” Journal of Nursing Management 28, no. 3: 514–521. 10.1111/jonm.12949. 31909847

[jan16791-bib-0009] Gungor, S. , and I. K. Tosunoz . 2024. “Levels of Nursing Students' Exposure to Colleague Violence and Affecting Factors: A Multicenter Cross‐Sectional Study.” La Medicina del Lavoro 115, no. 4: e2024024. 10.23749/mdl.v115i4.15606. 39189376 PMC11424089

[jan16791-bib-0010] Həsənli, S. 2024. “Analysis of the Psychological Characteristics of Mobbers Involved in Mobbing in an Enterprise.” Scientific Works 91, no. 1: 195–198. 10.69682/azrt.2024.91(1).195-198.

[jan16791-bib-0011] Hung, C. A. , P. L. Wu , N. Y. Liu , W. Y. Hsu , B. O. Lee , and H. C. Pai . 2019. “The Effect of Gender‐Friendliness Barriers on Perceived Image in Nursing and Caring Behaviour Among Male Nursing Students.” Journal of Clinical Nursing 28, no. 9–10: 1465–1472. 10.1111/jocn.14693. 30358000

[jan16791-bib-0012] IBM Corp . 2015. IBM SPSS Statistics for Windows. Armonk, NY: IBM Corp.

[jan16791-bib-0037] Johnston, S. , A. Fox , S. Patterson , et al. 2024. “Australian Nursing Students’ Experiences of Workplace Violence During Clinical Placement: A Cross‐Sectional Study.” Journal of Advanced Nursing 80, no. 12: 4933–4945. 10.1111/jan.16189. 38571292

[jan16791-bib-0013] Karatepe Kuscu, H. , U. Yuce Ozcan , and D. Atik . 2020. “Nursing Students' Opinions on Colleague Violence: A Qualitative Study.” Journal of Inonu University Vocational School of Health Services 8, no. 2: 219–232. 10.33715/inonusaglik.720000.

[jan16791-bib-0014] Kirbas, Z. Ö. , and İ. Kahriman . 2023. “Evaluation of Nursing Students' Exposure to Colleagues Violence in Application Areas.” Gumushane University Journal of Health Sciences 12, no. 2: 524–533. 10.37989/gumussagbil.1225590.

[jan16791-bib-0015] Kline, R. B. 2015. Principles and Practice of Structural Equation Modeling. 4th ed. New York: Guilford Press.

[jan16791-bib-0016] Koc, M. , and D. Batkin . 2016. “Exposure of Nursing and Midwifery Students to Colleague Violence in Practice Areas.” Anatolian Journal of Nursing and Health Sciences 19, no. 3: 189–196. 10.17049/ahsbd.11056.

[jan16791-bib-0017] Kuslu, S. , D. Ayar , C. Aksu , and B. Caki . 2022. “The Effect of Coronavirus Pandemic on the Nursing Image in the Society.” Eurasian Journal of Health Sciences 5, no. 3: 1–11. 10.53493/avrasyasbd.1014512.

[jan16791-bib-0018] Liu, W. , S. Zhao , L. Shi , et al. 2018. “Workplace Violence, Job Satisfaction, Burnout, Perceived Organisational Support and Their Effects on Turnover Intention Among Chinese Nurses in Tertiary Hospitals: A Cross‐Sectional Study.” BMJ Open 8: e019525. 10.1136/bmjopen-2017-019525. PMC6009508 29886440

[jan16791-bib-0019] Maffissoni, A. L. , M. d. S. Sanes , P. Bresolin , J. G. Martini , D. G. Schneider , and M. M. Lino . 2021. “Self‐Reported Violence by Nursing Students in the Context of Undergraduate Studies.” Revista Brasileira de Enfermagem 74: e20201179. 10.1590/0034-7167-2020-1179. 34287563

[jan16791-bib-0020] Mento, C. , M. Silvestri , A. Bruno , et al. 2020. “Workplace Violence Against Healthcare Professionals: A Systematic Review.” Aggression and Violent Behavior 51: 101381. 10.1016/j.avb.2020.101381.

[jan16791-bib-0035] Mohamed, F. B. M. , L. J. Cheng , X. E. C. Chia , H. Turunen , and H. G. He . 2024. “Global Prevalence and Factors Associated with Workplace Violence Against Nursing Students: A Systematic Review, Meta‐Analysis, and Meta‐Regression.” Aggression and violent behavior 75: 101907. 10.1016/j.avb.2023.101907.

[jan16791-bib-0021] Olgun, S. , and G. Kaptan . 2022. “Determination of Nursing Students' Perceptions of Nursing Image.” Health and Society 32, no. 1: 159–166.

[jan16791-bib-0022] Ozdelikara, A. , S. Agacdiken , and E. Aydin . 2016. “Nursing students' Choice of Profession and Factors Affecting It.” Acibadem University Journal of Health Sciences 2: 83–88.

[jan16791-bib-0023] Rudin, N. , and S. Ludin . 2018. “Mentorship Programme Criteria and Performance Outcomes of nurses' Perceptions.” Makara Journal of Health Research 22: 34–39. 10.7454/MSK.V22I1.8799.

[jan16791-bib-0024] Samadzadeh, S. , and M. Aghamohammadi . 2018. “Violence Against Nursing Students in the Workplace: An Iranian Experience.” International Journal of Nursing Education Scholarship 15, no. 1: 20160058. 10.1515/ijnes-2016-0058. 29373317

[jan16791-bib-0025] Stead, G. , L. LaVeck , and H. Rúa . 2021. “Career Adaptability and Career Decision Self‐Efficacy: Meta‐Analysis.” Journal of Career Development 49: 951–964. 10.1177/08948453211012477.

[jan16791-bib-0026] Sukut, O. , and C. H. Ayhan . 2022. “Nursing students' Experiences of Horizontal Violence and Occupational Belonging During Clinical Placements.” Journal of Integrative Nursing 4, no. 4: 231–238. 10.4103/jin.jin_15_22.

[jan16791-bib-0027] Tabachnick, B. G. , and L. S. Fidell . 2013. Using Multivariate Statistics. 6th ed. California: Pearson.

[jan16791-bib-0028] Tengiz, F. İ. , and A. B. Babaoglu . 2020. “Career Preferences of Senior Students in Faculty of Medicine and Factors Affecting These Preferences.” SDU Faculty of Medicine Journal 27, no. 1: 67–78. 10.17343/sdutfd.560350.

[jan16791-bib-0029] Tonkus, M. B. , and A. Coskun . 2021. “Determination of Nursing Department Students Exposure to Mobbing in Clinical Practice.” Journal of Turkish Nurses Association 2, no. 1: 15–26.

[jan16791-bib-0030] Totur Dikmen, B. , Y. Altinbas , O. Soyer Er , et al. 2022. “Effect of Perceptions of Professional Image on the Profession Preferences of Nursing Students: A Multi‐Centered Study.” Revista San Gregorio 1, no. 51: 15–32.

[jan16791-bib-0031] Wilson, J. L. 2016. “An Exploration of Bullying Behaviours in Nursing: A Review of the Literature.” British Journal of Nursing 25, no. 6: 303–306. 10.12968/bjon.2016.25.6.303. 27019166

[jan16791-bib-0032] Yilmaz Bahadir, E. , E. E. Ata , and G. Uyumaz . 2020. “Development of Colleague Violence Exposure Scale for Nursing Students.” Turkey Clinics J Med Ethics 28, no. 2: 188–198.

[jan16791-bib-0033] Yusupu, R. 2015. Relationships between career decisions and perfectionism, learning motivation and academic achievement in university students (Master's thesis, Institute of Educational Sciences).

