# Supplementary material for: Association Between Colleague Violence and the Professional Image of Nursing and Career Decisions Among Nursing Students: A Cross‐Sectional Study
Source: J Adv Nurs. 2025 Feb 3;81(10):6468–77. doi: 10.1111/jan.16791 (PMC12460971; doi:10.1111/jan.16791)
Supplement: Supplementary file 1 — Data S1. Supporting Information. [file JAN-81-6468-s001.docx]

**HISTOGRAM**

**ECVS**


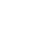

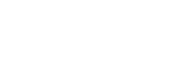

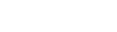

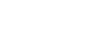

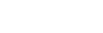

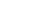

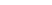

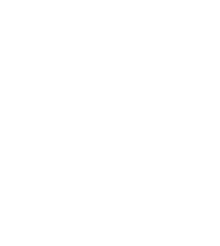

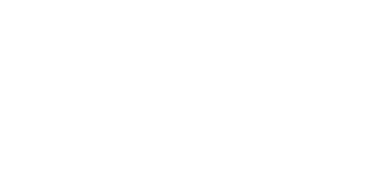

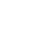


**
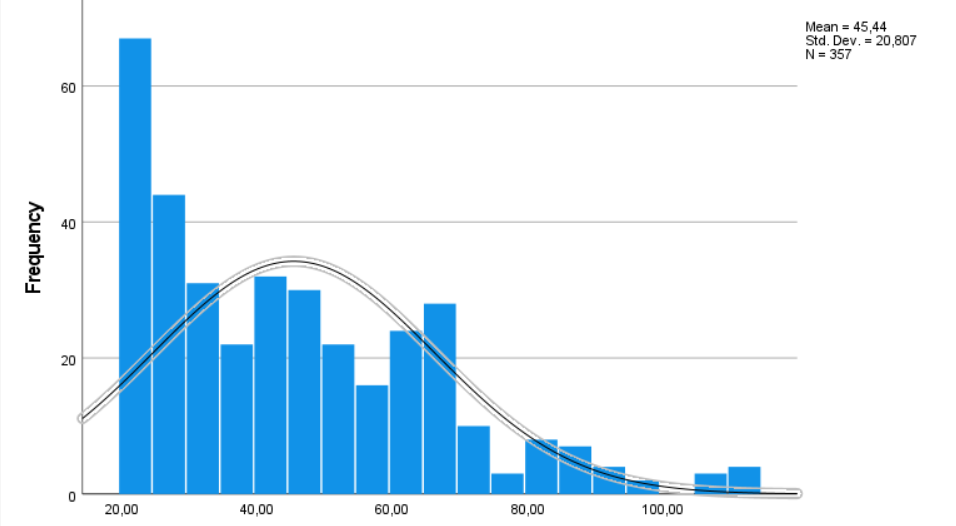
**

**INPS**

**
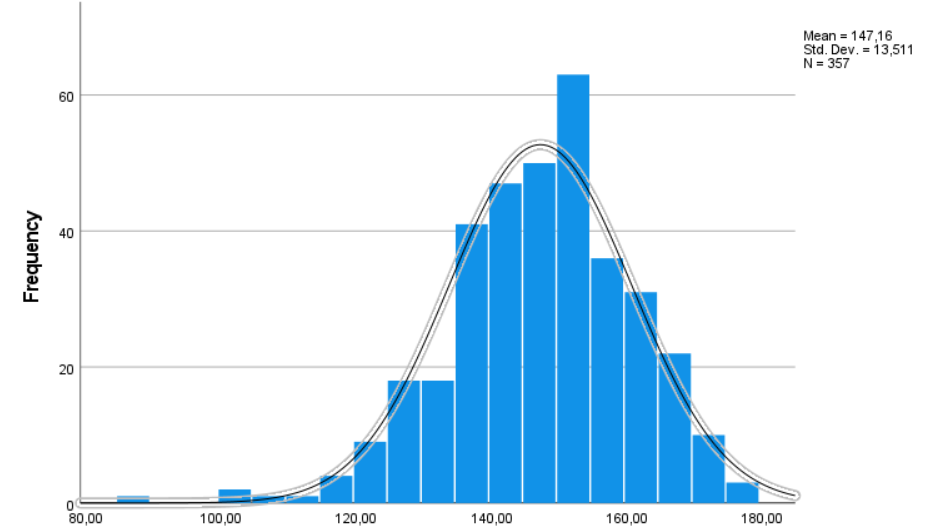
**

**CDS**

**
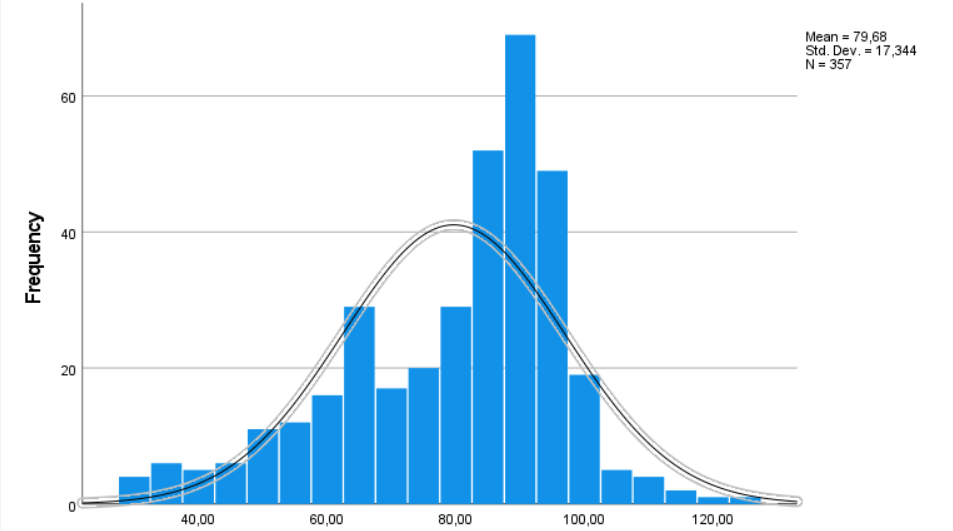
**

**SKEWNESS-KURTOSIS**

| **Descriptive Statistics** | | | | |
| --- | --- | --- | --- | --- |
|  | | | Statistic | Std. Error |
| ECVS | Mean | | 45,4370 | 1,10125 |
|  | 95% Confidence Interval for Mean | Lower Bound | 43,2712 |  |
|  |  | Upper Bound | 47,6027 |  |
|  | 5% Trimmed Mean | | 43,8413 |  |
|  | Median | | 43,0000 |  |
|  | Variance | | 432,949 |  |
|  | Std. Deviation | | 20,80743 |  |
|  | Minimum | | 22,00 |  |
|  | Maximum | | 110,00 |  |
|  | Range | | 88,00 |  |
|  | Interquartile Range | | 33,50 |  |
|  | Skewness | | ,887 | ,129 |
|  | Kurtosis | | ,314 | ,257 |
| INPS | Mean | | 147,1569 | ,71508 |
|  | 95% Confidence Interval for Mean | Lower Bound | 145,7505 |  |
|  |  | Upper Bound | 148,5632 |  |
|  | 5% Trimmed Mean | | 147,5380 |  |
|  | Median | | 148,0000 |  |
|  | Variance | | 182,548 |  |
|  | Std. Deviation | | 13,51105 |  |
|  | Minimum | | 87,00 |  |
|  | Maximum | | 178,00 |  |
|  | Range | | 91,00 |  |
|  | Interquartile Range | | 17,00 |  |
|  | Skewness | | -,548 | ,129 |
|  | Kurtosis | | 1,091 | ,257 |
| CDS | Mean | | 79,6751 | ,91794 |
|  | 95% Confidence Interval for Mean | Lower Bound | 77,8698 |  |
|  |  | Upper Bound | 81,4803 |  |
|  | 5% Trimmed Mean | | 80,5003 |  |
|  | Median | | 85,0000 |  |
|  | Variance | | 300,815 |  |
|  | Std. Deviation | | 17,34403 |  |
|  | Minimum | | 30,00 |  |
|  | Maximum | | 123,00 |  |
|  | Range | | 93,00 |  |
|  | Interquartile Range | | 24,50 |  |
|  | Skewness | | -,794 | ,129 |
|  | Kurtosis | | ,246 | ,257 |
